# What do we want to know about MOOCs? Results from a machine learning approach to a systematic literature mapping review

**DOI:** 10.1186/s41239-022-00359-1

**Published:** 2022-10-14

**Authors:** Ignacio Despujol, Linda Castañeda, Victoria I. Marín, Carlos Turró

**Affiliations:** 1 Universitat Politècnica de València, Valencia, Spain; 2 Universidad de Murcia, Murcia, Spain; 3 Universitat de Lleida, Lleida, Spain

**Keywords:** MOOCs, Machine learning, Clustering

## Abstract

By the end of 2020, over 16,300 Massive Open Online Courses (MOOCs) from 950 universities worldwide had enrolled over 180 million students. Interest in MOOCs has been matched by significant research on the topic, including a considerable number of reviews. This study uses Machine Learning techniques and human expert supervision to generate a comprehensive systematic literature mapping review that overcomes some limitations of the traditional ones and provides a broader overview of the content and main topics studied in the specialized literature devoted to MOOCs. The sample consisted of 6320 publications automatically classified within six research topics, denominated by human experts: institutional approach, pedagogical approach, evaluation, analytics, participation, and educational resources. The content analysis of the topics identified was conducted using visual network analysis, which supported the identification of different thematic sub-clusters and endorsed the classification. Results from the review show that the lowest production of MOOC papers is within the topics of the pedagogical approach and educational resources. In contrast, participation and evaluation are the most frequent ones. In addition, the most cited papers are on the topics of analytics and resources, being the pedagogical approach and the institutional approach the less cited. This highlights the need for more MOOC research from a pedagogical perspective and calls upon the presence of educators.

## Introduction

Massive Open Online Courses (MOOCs) is a term coined in 2008 by David Cormier and Bryan Alexander to name the experience created when Stephen Downes and George Siemens launched their course 'Connectivism and Connective Knowledge/2008' (CCK8) and worked and learned actively with 2200 people (Siemens, 2012). But more than a term, MOOCs are a phenomenon that represents one of the most influential initiatives in Higher Education, aiming to adopt and use effectively the most popular digital technologies' features, all of which with more than a decade of history (Rodriguez, 2012).

This influence in the day to day of universities all over the world –at the end of 2020 there were over 16,300 courses from 950 universities worldwide and more than 180 million enrolments (Shah, 2020)—, and the amazing data generated by the experiences—precisely because of the massive character of the courses—, has fostered the generation of a variety of studies and analysis about their implementation, and a vast amount of literature on the topic. Illustratively, by the end of 2020, over 4000 records included the keyword MOOC in their title or abstract in the Web of Science (WOS) database, and more than 5100 records did it in the Scopus database. Therefore, the term MOOC has been the object of study of an impressively high number of literature reviews ranging from 2013 (Liyanagunawardena et al., 2013), until more recent times (Babori, 2020).

Nevertheless, despite their conclusions being interesting and valuable, and given the vast amount of existing literature, the sample size of those reviews, the sampling mechanisms used in systematic literature reviews and other of their traditional limitations (Davies, 2000; Widiger et al., 1990), the conclusions obtained from these literature reviews remain partial and is very difficult to generalize knowledge about what the specialized literature says about what has been already studied about MOOCs.

In this paper, we propose an alternative approach to a literature review that, using machine learning (ML) and Visual Network Analysis (VNA) techniques, provides researchers and stakeholders with a realistic overview of the content and main topics studied in the specialized literature regarding MOOCs published and indexed in two of the main databases (WOS and SCOPUS) until the end of 2020. This overview aims at describing the produced literature about MOOCs by highlighting the main group of topics that can be found in the published studies (clusters), their relevance and impact and the influence between them. It helps to highlight the priorities these studies had and the topics they did not address. With these two techniques, we aim at offering a complementary and more comprehensive vision to previous literature reviews on MOOCs, looking at the whole picture of what has been published.

## Literature reviews regarding MOOCs

MOOCs have sparked an outstanding interest among the educational technology research community. Acknowledging the number of studies already conducted on MOOCs, many authors have also conducted very interesting literature reviews to map what we already know about MOOCs.

The previous literature reviews on MOOCs have covered very diverse sample sizes of publications (ranging from less than 10 to over 300 publications), different publication periods and ranges (from 2 to 9 years) and varied findings regarding what is already known about MOOCs. Most of these reviews analyzed MOOCs' origins, their research method and collection of data (qualitative or quantitative studies), and the most frequent topics. Some include a classification of topics or groups of topics in the papers analyzed.

To provide an overview of some of these literature reviews and their specific findings, they have been synthesized in Table 1.

**Table 1:** Synthesis of literature reviews on MOOC research

Review	N	Period	Findings
Babori (2020)	100 papers	2012–2018	Four categories of research were identified: (1) learning process (39%), (2) predictors of retention (17%), (3) learning experiences (21%) and (4) design of MOOCs (23%). 45% of the articles did not have an identifiable theoretical framework, and the rest of the frameworks were centered on learning analytics
Bozkurt et al. (2017)	362 papers	2008–2015	Three research areas out of 15 concentrated more than half of the research, most articles focused on xMOOCs, and their discourse is mostly neutral (56%). However, articles with a positive outlook (27%) outweighed those that are negative (1%) or critical (16%)
Bozkurt et al. (2016)	51 theses and dissertations	2008–2015	Education, engineering and computer science and information and communication technology are the main disciplines within MOOC research. Qualitative methods were preferred, and half of them did not have a theoretical framework, the documents studied mainly xMOOCs and focused mainly on MOOC learners and MOOC systems with an educational perspective
Deng and Benckendorff (2017)	53 papers	2014–2016	Most articles used only one research method. Surveys, interviews, and log files extracted from MOOC platforms were the most common sources of information, with diary studies and focus groups being less common
Ebben and Murphy (2014)	25 papers	2009–2013	It distinguishes two MOOC development phases: one focused on connectivism and a second one based on xMOOC rise and development
Hew and Cheung (2014)	25 papers	––	Motivations and challenges of using MOOCs by students and instructors were studied, trying to identify issues not fully addressed or resolved
Kennedy (2014)	6 papers	––	Key characteristics of MOOCs: varied definitions of openness, barriers to persistence with a high dropout rate and a distinct structure with two pedagogical approaches, XMOOCs and CMOOCS
Liyanagunawardena et al. (2013)	45 papers	2008–2012	Eight categories: introductory, concept, case studies, educational theory, technology, participant focused, provider focused, and other
Raffaghelli et al. (2015)	60 papers	2008–2014	Nine research aims: Methodological approaches to study MOOCs, Literature review, Institutional development, Teaching processes, Technological tools, Pedagogy, Contribution to educational theory, Learning processes, Learning design
Rasheed et al. (2019)	311 papers	2009–2018	MOOC research is done mainly in the United States and a few European countries. Most of the studies used quantitative (53%) or mixed (30%) research methods and used one data collection method (75%) They also identified 18 key topics (addressing learners' completion/dropout/retention was the most popular with a percentage of 12.9%)
Sa'don et al. (2014)	164 papers	2008–2014	10 nascent research trends in MOOC research, ordered by their relevance: Pedagogy, Assessment and accreditation, Engagement or motivation, Knowledge sharing, Cultural diversity, Technology, Social Interaction, Participant retention, Learning analytics and Policy and Instructional design
Sangrà et al. (2015)	228 papers	2013–2014	The authors identified 11 areas and found that Pedagogical strategies, Student engagement and motivation, the Role of social networks in teaching and learning and Consequences for Higher Education systems were the most popular focus areas
Veletsianos and Shepherdson (2016)	183 papers	2013–2015	They studied geographic distributions of the authors, publication outlets (journals or conference proceedings), data collection and analysis methods (with 8 categories for data collection and 11 categories for data analysis), citations on Google Scholar and research strands (student-focused, teacher-focused, design focused, context and impact, other)
Yousef et al. (2014)	84 papers	2008–2013	It classifies papers in 7 dimensions: concept, design, learning theories, case studies, business model, targets groups, and assessment
Zhu et al. (2018)	146 studies	2014–2016	Most studies used quantitative research methods (46%), followed by mixed research methods (36%). Among the foci of that research, learner retention and motivation were the most mentioned, followed by learner experience and satisfaction, assessment, and instructional design. They also identified 24 key topics

All these reviews provide very valuable conclusions for the field, but they have a common limitation, the size of their samples. Eight out of the 15 reviews in the table work with a sample of fewer than 100 papers. The wider one was the one made by Rasheed et al. (2019), that studied 311 papers from 2009 to 2018. Although most use the human potential and expertise of their authors to analyze and attempt to consolidate a classification of the literature on MOOCs, the low representativeness of these samples, compared to the entire corpus published on the subject, makes most of them more a sampling of the interests of MOOC researchers around the world than a complete picture of MOOC research.

## Research questions

This research aims to analyze MOOC-related publications appearing in specialized databases since the emergence of the term associated with education, using ML and VNA techniques. In doing this, the study focused on exploring four main aspects corresponding with the main research questions, as follows:

- RQ1: What groups of topics (Thematic Clusters) can we identify in the literature studied using ML techniques?
- RQ2: How can we characterize each thematic cluster based on the relationships established between the terms it handles (SNA)?
- RQ3: What relationships of relevance, impact or influence can we identify between the different thematic clusters?
- RQ4: What is the missing MOOC research?

## Methodology

### Sample and procedure

To answer those specific research questions, a systematic literature review has been conducted. Gough et al. (2017) state that traditional systematic reviews involve three key activities: identifying relevant research, critically reviewing the identified research reports in a systematic manner that can be reproduced, and synthesizing research findings to guide researchers in planning future studies.

Considering the popularity and considerable research on MOOCs over the years, using a systematic review approach can summarize all research and help to identify research gaps to move the MOOC research forward. This study specifically applies a mapping approach or "systematic mapping", since it focuses on describing the research field rather than synthesizing findings (Newman & Gough, 2019).

To collect the sample, the first step consisted of a simple query for the keyword "MOOC" in the title, abstract and keywords fields in two of the most relevant literature databases over the world: Web of Science (WOS), and SCOPUS (as of December 19, 2020). These two databases are considered the most widely used reference databases in the scientific and academic fields (Archambault et al., 2009) and are reliable to be considered principal systems for systematic reviews (Gusenbauer & Haddaway, 2020).

The results were downloaded in CSV (comma separated values) format files and joined in an Excel data model. As in any machine learning project, data understanding and data preparation phases were needed before applying the machine learning algorithms (Mayo, 2018).

The data was consolidated in a single table with columns for the year, title, abstract, and authors. After analysing the data in the data understanding phase, a couple of issues were detected for the data preparation phase: first, MOOC acronym is used in Optics and Oxide field with other purposes, so a filter was applied with these two words in the abstract and all the non-relevant articles were eliminated. And second, the duplicated articles in the two databases sometimes contained slight differences in titles, and abstracts (for example different number of spaces, some differences in the capitalization of letters or different punctuation symbols) that made automatic elimination of duplicates by comparison imperfect, so extra fields were prepared with the 100 first characters in lower case from title and abstract fields, after eliminating spaces and punctuation symbols, to use them with the eliminate duplicates function of excel. Finally, the table was ordered alphabetically by the title field and a manual revision of the resulting table was conducted to detect duplicates not eliminated by the former processes. The table ended with 6320 rows.

Different reviews have already been conducted on MOOCs. However, the particularity is that Machine Learning (ML) algorithms (unsupervised machine learning) have been used instead of manually identifying research and reviewing it, to see if the obtained results are like the ones from former reviews done manually and, therefore, if systematic reviews can be optimized in time and resources.

Unsupervised machine learning is a category of ML that includes algorithms that learn patterns from untagged data (Sarker, 2021). Extracting topics is a good unsupervised data-mining technique to discover the underlying relationships between texts, so a technique called Topic Modelling of Natural Language Processing (NLP) was applied. Topic Modelling is a special type of clustering algorithm that makes the clusters dependent on distributing the vocabulary and extracts the characteristics of each cluster, as described by Ahmed et al. (2021). As in some clustering algorithms, these algorithms need the user to specify the number of different clusters to be created. This number can be chosen manually or can be selected using a coherence score technique as explained by Röder et al. (2015). The idea is to calculate the coherence score of the models created with different clusters and select the one with the highest coherence. With these ML algorithms, a list of different groups of articles (thematic clusters), characterized by their most representative words, was created, and each article was assigned to one of these clusters.

With the most relevant divisions made by the ML method, an expert focus group was carried out to select the most meaningful one, according to educational criteria. A Focus group, in this case, a mini-focus group (Scholz, 2001) was introduced in this study to introduce expert knowledge and opinions from a group of key informants (Payne & Payne, 2004).

In this study, focus group participants, based on their expertise, analyzed the different divisions made by the ML method and, introducing an abductive reasoning process (Flick, 2017), discussed and agreed on which of them was the best option among the one proposed.

Four academics, experts in Educational Technology and Higher Education (2 male and 2 female), from two different European countries, were invited to an online face-to-face discussion via Zoom. The four of them received the materials (the different clusters divisions made by the ML with a list of the keywords included on each cluster) with a short description of the method used to get the clusters and a brief introduction to what were the next steps to carry out in the process. During the meeting, some questions about the ML process were solved, and the consensus was arrived after 45 min.

**Fig. 1:**
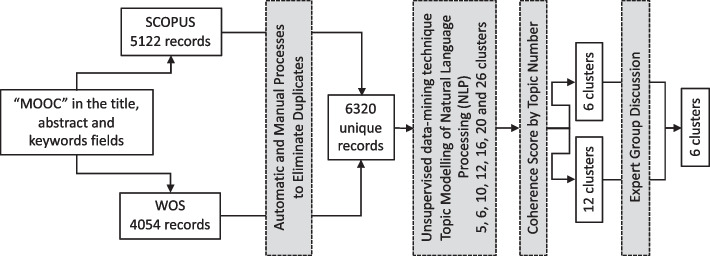
Sampling process

The whole process followed by the sampling methodology described above is shown in Fig. 1.

For the content analysis of the thematic clusters, we used as a method the Visual Network Analysis (VNA), which supports the visualization of the dynamics of networks and their components and focuses on the visual characteristics of networks for a qualitative interpretation (Decuypere, 2019), which differs from Social Network Analysis (SNA) (Wasserman & Faust, 1994), that focuses on the structural social properties of networks. Titles and abstracts were analyzed using the text-mining functionality of VOSViewer 1.6.11 to construct and visualize co-occurrence networks of the most prominent terms from the literature (van Eck & Waltman, 2010), after manually deleting similar and non-significant words through the use of a thesaurus created and iteratively developed by the experts. Considering a manageable and meaningful size, so humans can interpret the groups, and after different tests, terms with a threshold of 20 appearances—except from cluster 5, which had fewer words to show, so the threshold was lowered to 15 appearances—were set to be included in the visualization of each group. This should be also acknowledged as a limitation, as terms with less than 20 appearances in each group are not shown in the visualizations. With the number of words corresponding to that condition, VOSViewer calculates a relevance score to show only the 60% most relevant terms—this automatic process differentiates between noun phrases with a general meaning and with a specific meaning (van Eck & Waltman, 2011).

Also, to analyze the clusters' relevance, influence, and impact, we have analyzed the main statistics of the papers on each cluster, using the data provided by the WOS and SCOPUS (e.g., number of citations). The authors of this paper did the interpretation of visualizations.

### Results

Our final sample included 5122 records from SCOPUS and 4054 records from WOS. After eliminating duplicates, these records were condensed into a sample of 6320 resources, including journal papers, conference papers, editorials, etc.

From these 6320 documents, more than a half are conference papers (n = 3722, 58.89% of the sample- including long, short and specific papers published in proceedings), a big portion of them are journal papers (n = 2282, 36.11%). The lowest numbers correspond to book chapters (n = 213, 3.37%) and full books (n = 14). There are 74 reviews dedicated to MOOCs during the studied period.

**Fig. 2:**
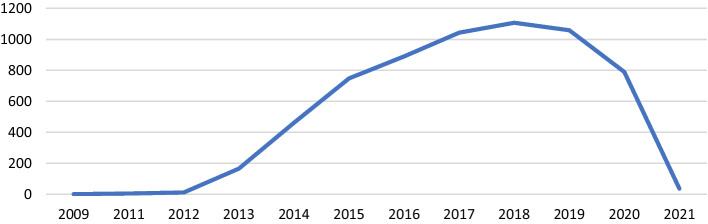
Distribution of papers over the years (n = 6320)

Over the years, distributing the papers shows the evolution of the interest in the topic and the intense hype lived by the topic since 2015, and the stabilization around 2018/2019 (see Fig. 2).

The coherence score of the clustering analysis was done with one to fifty groups, obtaining Fig. 10, which can be seen in Appendix A.

In Appendix Fig. 10 the coherences for 6 and 12 groups are the biggest ones, with 6 groups having a slightly higher coherence, which is indicated in the title of the figure and can be seen comparing with the red line drawn to mark the peak coherence.

Six groups and 12 groups were selected as the most interesting classifications (see Appendix B). The coherences obtained are the same every time the algorithm is run.

The classification with 6 groups has a slightly higher coherence, but the difference with 12 groups is very small. The final decision about the number of clusters to use in the analysis was entirely based on the educational perspective of the 4 experts on e-learning in Higher Education. The relevance of the combination of machine learning and human interpretation with expertise in the area is essential in order to make sense of the data obtained. They discussed which division of groups would give a more educational approach to analyzing the MOOCs literature. The classification with 6 groups was the one chosen.

A mapping review of each thematic cluster and a comparison between them to understand how they classify the literature is presented below. In this part, two of the four experts worked first individually, to provide a title to each thematic cluster, and then put together their proposal and unified their suggestions without finding major discrepancies. After this phase, the two experts worked on the description of each cluster and discussed the sub-clusters and interpretations. A clear limitation of this process is the possible differences in interpretation of each of these thematic clusters, which we acknowledge. Visualizations greatly differ from each other, based on the number of publications included in each cluster, as well as the weight of the terms (the higher the number and strength of links, the more prominent and denser the terms are).

### Thematic cluster 1: institutional approach

This thematic cluster is characterized by the words: *education, university, course, development, technology, platform, institution, quality, country, world, opportunity, paper, challenge, access, MOOC*.

**Fig. 3:**
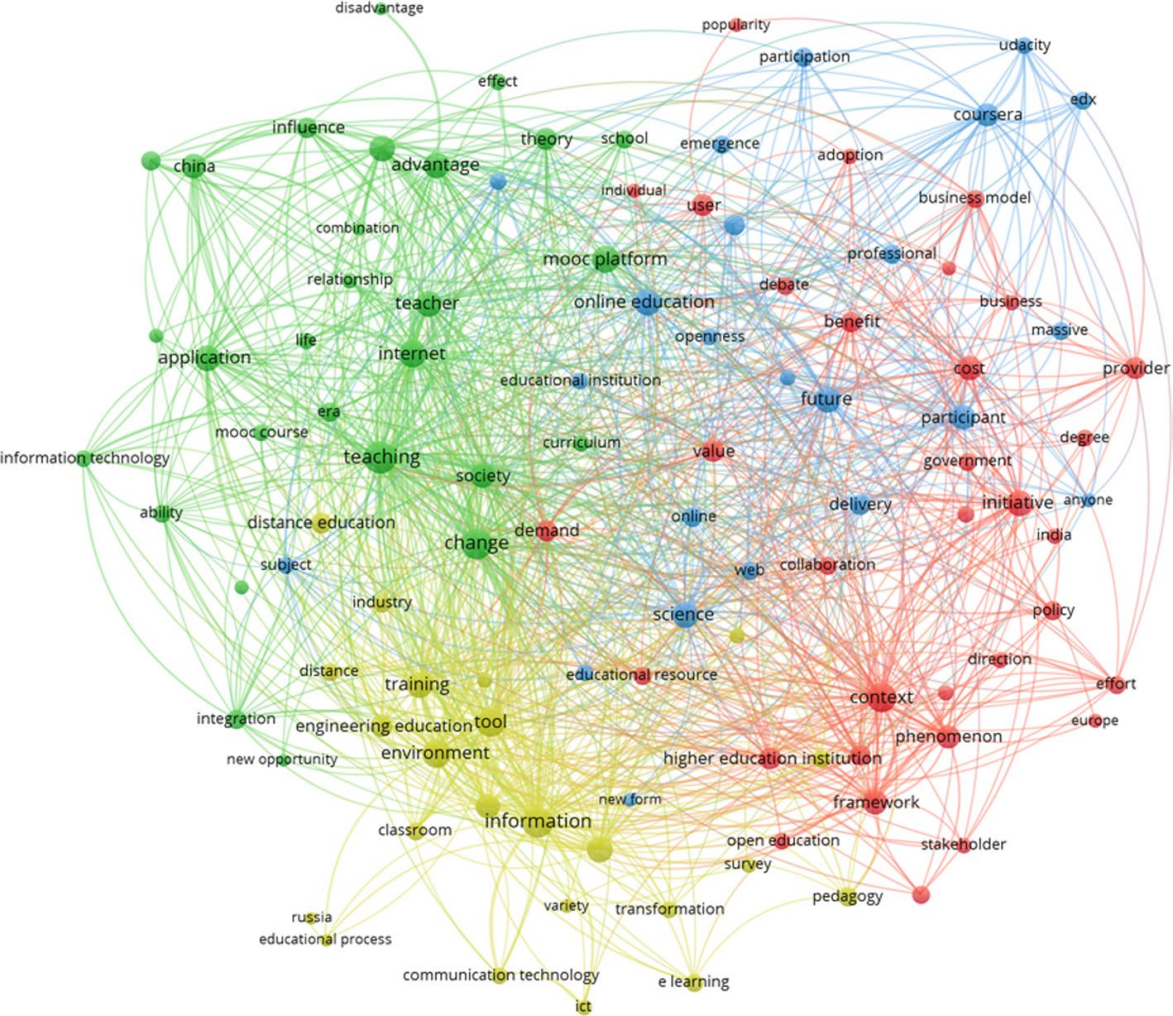
Thematic cluster institutional approaches about MOOCs. Network map

The representation of the network map of this cluster (see Fig. 3) has a clear center on *online education* (sub-cluster 3, blue—center-upper part). It shows us a very dispersed interest but with some clear sub-clusters that reinforce the institutional studies and approaches concept.

Five sub-clusters are identifiable. The red one (sub-cluster 1) combines the business perspective (with words such as e.g., *business model, benefit, cost, effectiveness, provider*) and the institutional perspective (*policy, government*), with the relevant presence of higher education. Terms that appear are *higher education institution, faculty, policy, government, stakeholder*, but also MOOCs as an online format (e.g., *open educational resources, open education, online learning*). The green one (sub-cluster 2) highlights the relations with education (e.g., *teacher, teaching, curriculum, school*) and a new life context (e.g., *society, life, change, era*). The blue one (sub-cluster 3) focuses on the open format of MOOCs (e.g., *anyone, massive, participant, participation, open access, openness*) and the platforms for delivery (e.g., *coursera, edx, udacity*). Finally, the yellow one (sub-cluster 4) is about the technological means and spaces from an educational perspective (e.g., *environment, tool, educational process, pedagogy*), the relation to *e-learning* and *distance education*, also interesting is the appearance of the term *transformation*. Some continents and countries can be identified in several sub-clusters, which may be key locations for MOOCs from an institutional viewpoint: *Europe* and *India* in sub-cluster 1, *China* in sub-cluster 2 and *Russia* in sub-cluster 4.

### Thematic cluster 2: pedagogical approach

**Fig. 4:**
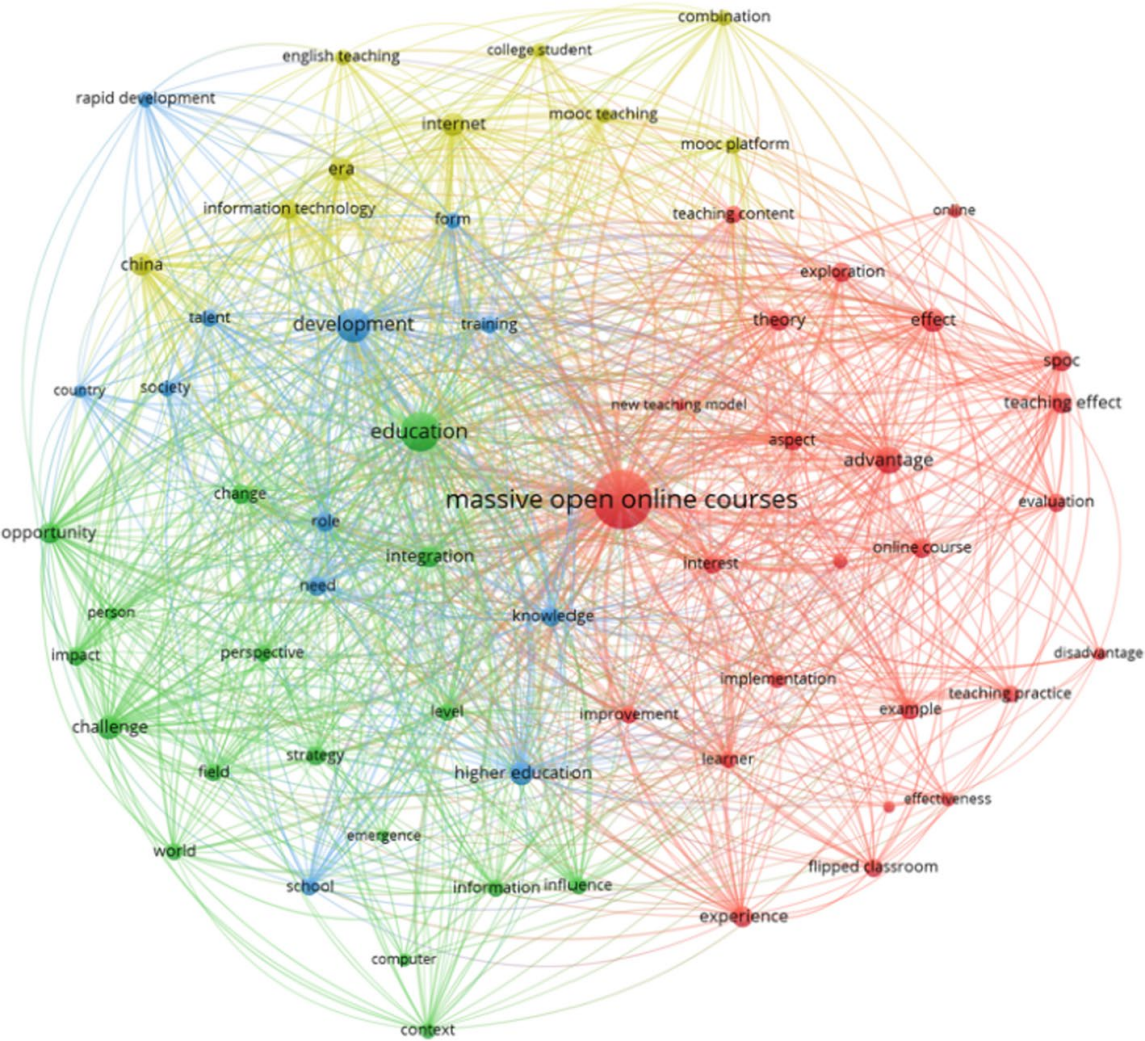
Thematic cluster pedagogical approach of MOOCs. Network map

This cluster is shaped by the terms: *teaching, mode, college, classroom, reform, model, method, teacher, application, ability, resource, computer, learning, curriculum, and effect* (see Fig. 4).

In the network map of this group, there is a clear center: *massive open online courses*, which is part of the sub-cluster 1 (red) from the 4 clear sub-clusters included on the group. The red sub-cluster 1 includes the application of MOOCs in education contexts, especially from a teaching perspective (e.g., *new teaching mode, new teaching model, teaching content, teaching practice, teaching process, teaching effect, learner*). The green one (sub-cluster 2) mentions the possibilities of MOOCs (e.g., *change, emergence, impact, opportunity*), and *education* is prominent. The blue one (sub-cluster 3) addresses the connection of MOOCs between institutions and professional training (e.g., *development, higher education, rapid development, school, society, talent, training*). The yellow one (sub-cluster 4) is a rather dispersed sub-cluster with words such as *mooc platform, mooc teaching, college student, information technology, internet*, interestingly the term *China* appears, which leads to a guess regarding the importance of MOOCs in that country. All the four sub-clusters remark the pedagogical character of the papers on this group.

### Thematic cluster 3: evaluation

**Fig. 5:**
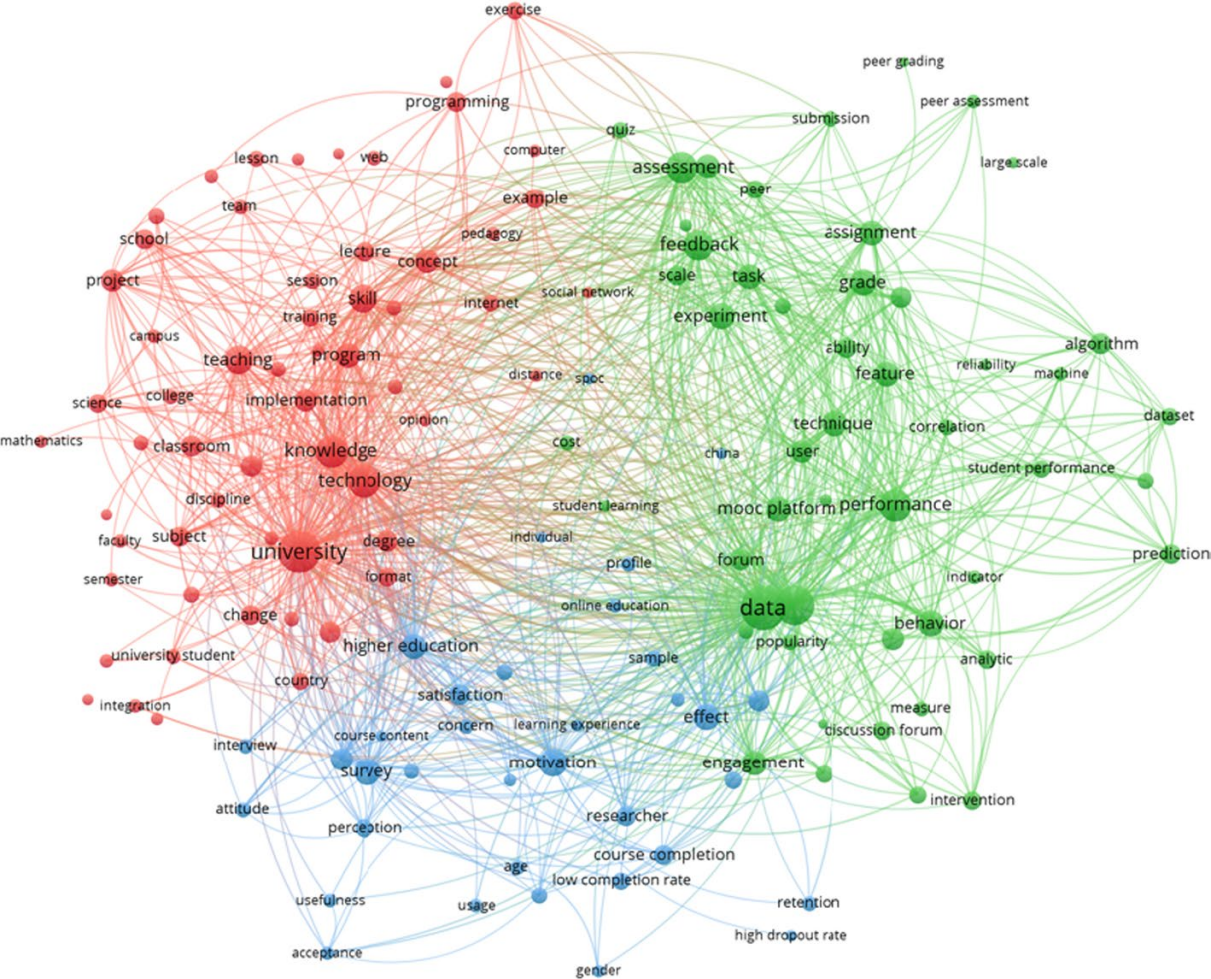
Thematic cluster evaluation of MOOCs. Network map

Cluster 3 is defined by terms such as *student, course, assessment, performance, study, datum, peer, result, feedback, engagement, group, programming, forum, rate, activity* (see Fig. 5).

On the network representation of this cluster, a focused distribution on three sub-clusters can be identified. However, there is no clear center on it, but instead two preponderant centers: *university* (sub-cluster 1, red) and *data* (sub-cluster 2, green). In the red, sub-cluster 1, more terms are related to university (e.g., *teaching, knowledge, degree, program, lecture, college, classroom*…). *School* is in the periphery, also *programming* is highlighted, and interestingly, *change* appears but not prominently. The green one (sub-cluster 2) includes terms related to the data *sources* and their uses, such as *performance, behavior or engagement*. Other terms are related to the data processes: *algorithm, accuracy, prediction, reliability*. Remarkable terms are related to assessment (upper part): *assessment, feedback, assignment, grade*. In the blue one (sub-cluster 3) the term *higher education* stands out. It shows research done with *survey* (more prominent) and *interview* (lower presence) regarding *perception, attitude, satisfaction, motivation, usefulness, acceptance or usage*. Again, *China* appears in this sub-cluster 3.

Additionally, the relations between the green sub-cluster and the blue one for data, *course completion, low completion rate, retention, high dropout rate, effect* are very interesting.

### Thematic cluster 4: analytics

**Fig. 6:**
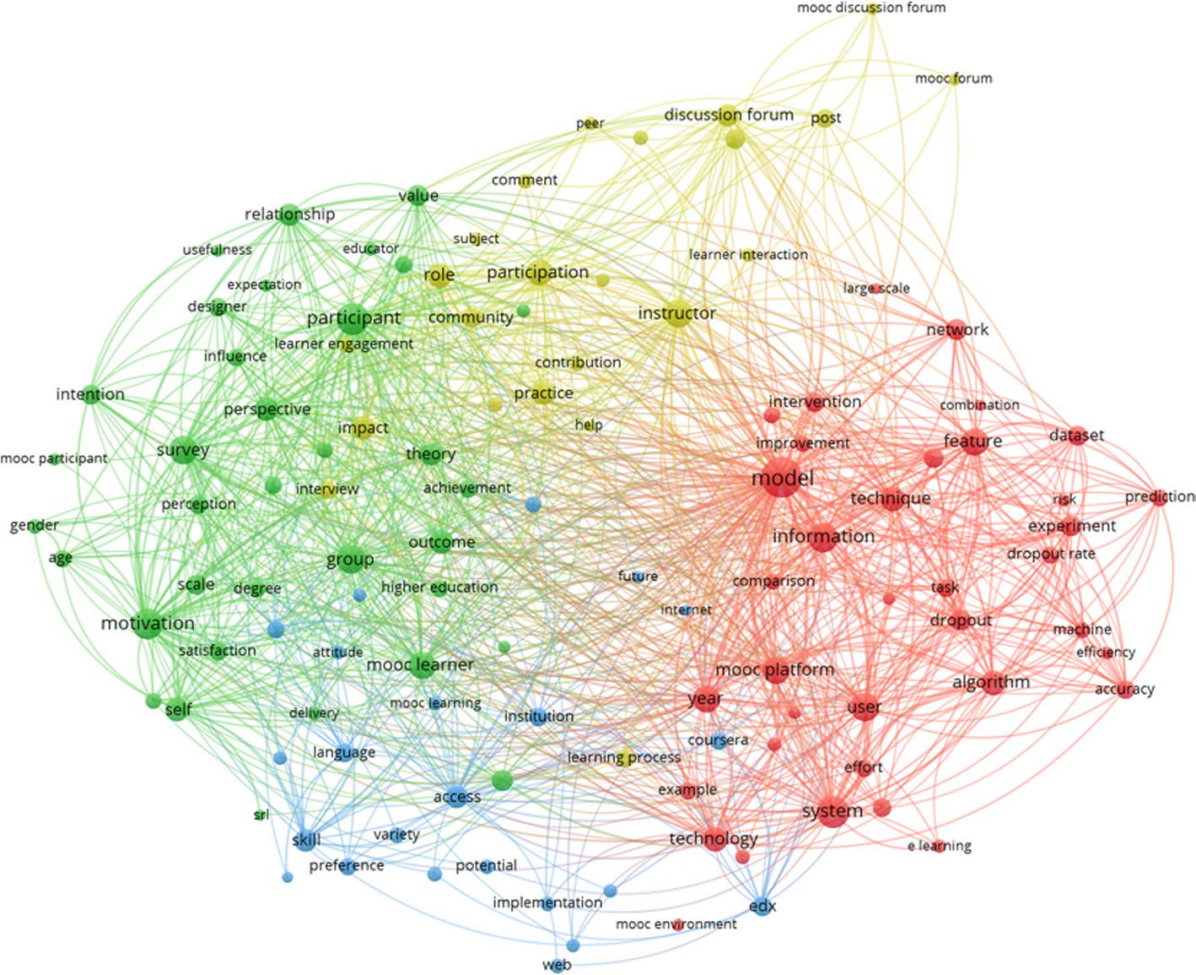
Thematic cluster analytics of MOOCs. Network map

The thematic cluster 4 is shaped by the terms: *learner, course, datum, learning, engagement, study, rate, behavior, forum, self-interaction, completion, dropout, analysis, motivation* (see Fig. 6)*.*

In this cluster map, there is not a clear center, and 4 sub-clusters have been defined. The red, sub-cluster 1, is about mathematical models to study MOOC large scale data (e.g., *model* (prominent), *information, technique, experiment, algorithm dropout, mooc platform*). The green, sub-cluster 2, shows as prominent elements *participation, perspective, survey, motivation, group, mooc learner and intention*, so it is related to studies on MOOC participants and their motivations to do so. In the blue, sub-cluster 3, it is difficult to clearly identify themes, but it seems related to MOOC implementation and teaching (e.g., *instruction, individual, preference, web*). Finally, in the yellow, sub-cluster 4, the terms consider MOOC activities and tasks: *assignment, course content, forum, participation, pedagogy, learning process*.

### Thematic cluster 5: participation

**Fig. 7:**
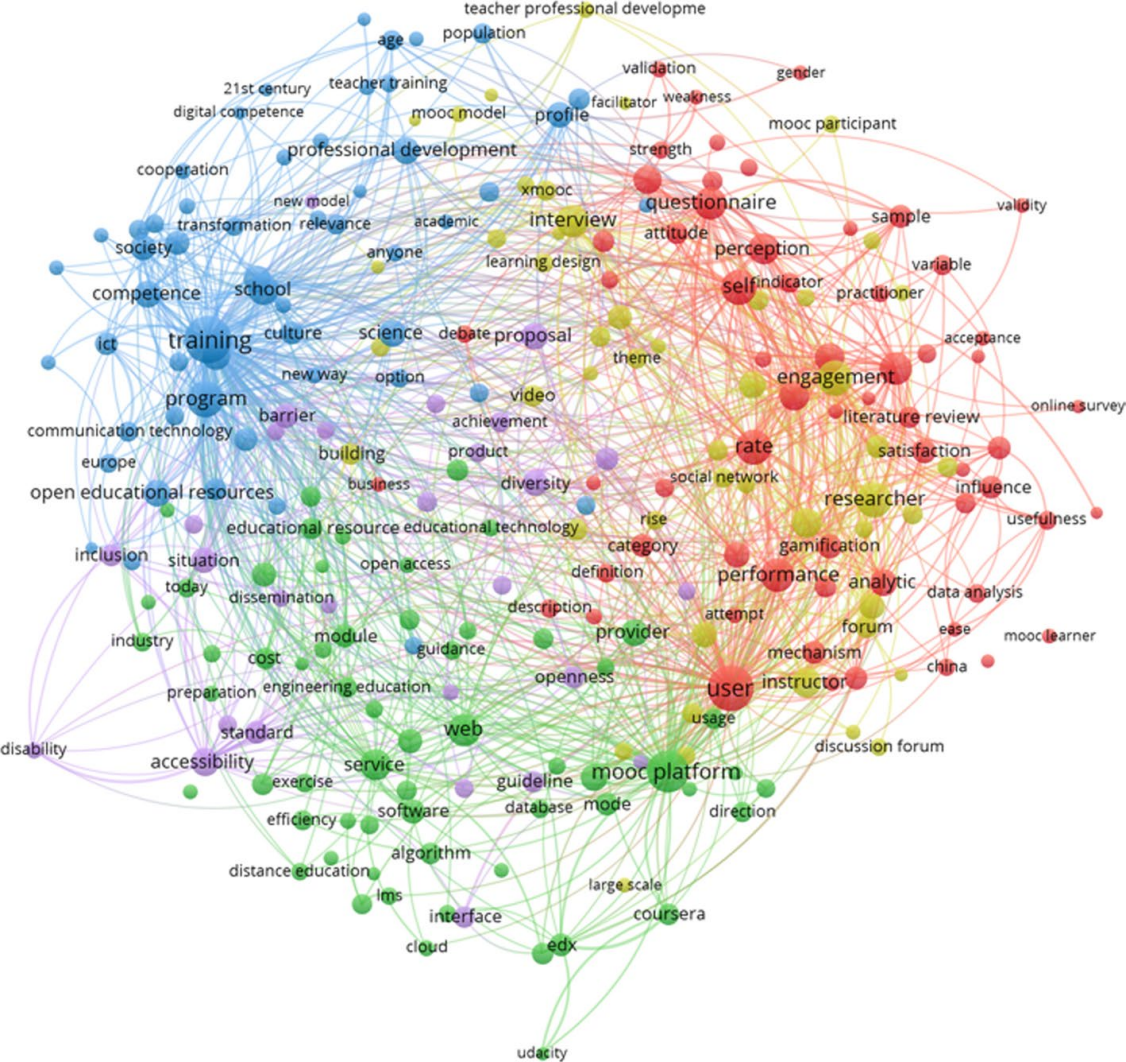
Thematic cluster participation on MOOCs. Network map

Cluster 4 is defined by: *learning design, teacher, research, study, environment, participant, process, development, tool, approach, experience, framework, project and community* (see Fig. 7).

In this cluster, there are no centers; nevertheless, the prominent terms on each sub-cluster provide many clues about the important topics of the papers included.

The yellow, sub-cluster 1, relates to user perceptions regarding MOOCs (e.g., *questionnaire, perception, engagement, performance, user, satisfaction*). The green, sub-cluster 2, may concern the link of MOOCs to prepare for technological training by combining words regarding education (e.g., *educational material, educational process, educational resource, lecturer*), *industry* and *computer science* (e.g., *programming*). In the blue, sub-cluster 3, *training* is prominent and connects to professional training (e.g., *program, professional, competence, open educational resources, professional development, lifelong learning, job, digital competence*). Geographical locations, such as *Mexico, Spain, and Europe,* could reveal the importance of this aspect in those places. The yellow, sub-cluster 4, includes aspects related to learning activity and *engagement* (prominent term) (e.g., *collaborative learning, course design, course material, discussion forum, facilitator, learning activity, learning design, MOOC environment*). Also, *cMOOC*, as a MOOC format with an increased participant engagement, appears. Finally, remarkably, fuchsia, sub-cluster 5, includes two new terms to the analysis: *accessibility and inclusion*.

### Thematic cluster 6: educational resources

**Fig. 8:**
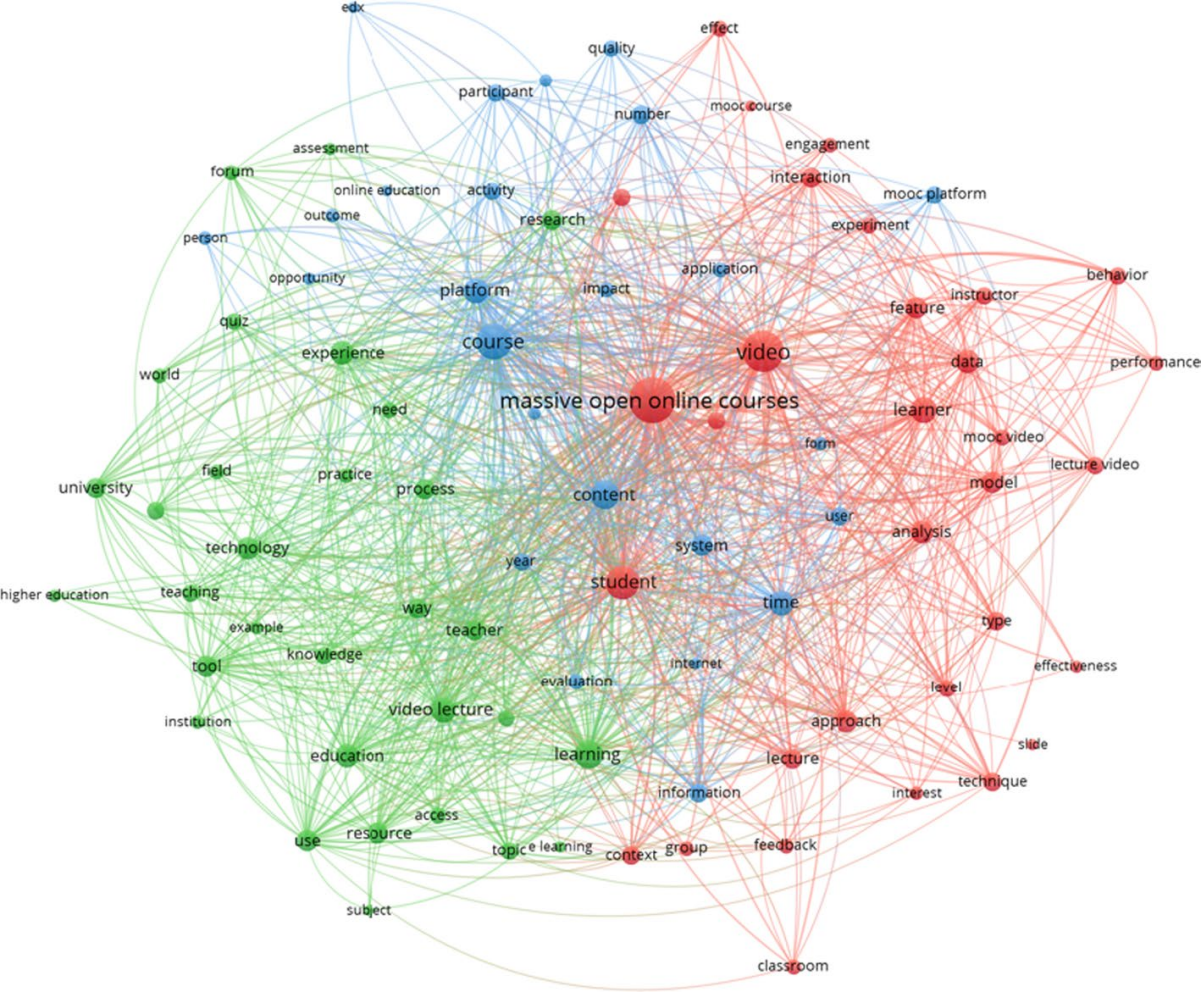
Thematic cluster educational resources for MOOCs. Network map

The last cluster of papers is defined by the terms: *video, lecture, content, style, user, feature, resource, course, production, quiz, material, time, platform, topic, behavior* (see Fig. 8).

In this map, the three sub-clusters are clear. The red, sub-cluster 1, includes the study of the resources, with special emphasis on the *video*, regarding the participants and the learning (e.g., *behavior, effect, effectiveness, engagement, interaction, interest)*. The green, sub-cluster 2, concentrates papers focused on different aspects of teaching and learning in MOOCs, such as *assessment, course, education, higher education, teacher and teaching*. Finally, the blue, sub-cluster 3, is shaped by diverse aspects, such as *course, educational video, online education, quality or impact*.

## Cluster comparison

The most popular thematic clusters in the literature about MOOCs are related to participation on MOOCs, representing a third (33.88%) of the literature, and evaluation, with a 20.05% of the sample (see Table 2). Remarkably, the less popular topics are those related to the MOOC's pedagogical approach -with only 9.73% of publications- and papers related to resources to implement MOOCs, with just 5.52% of the literature production.

**Table 2:** MOOCs literature thematic Groups: production of papers

	Institutional approach	Pedagogical approach	Evaluation	Analytics	Participation	Resources
N	928	615	1267	1020	2141	349
Percentage	14.68	9.73	20.05	16.14	33.88	5.52

**Fig. 9:**
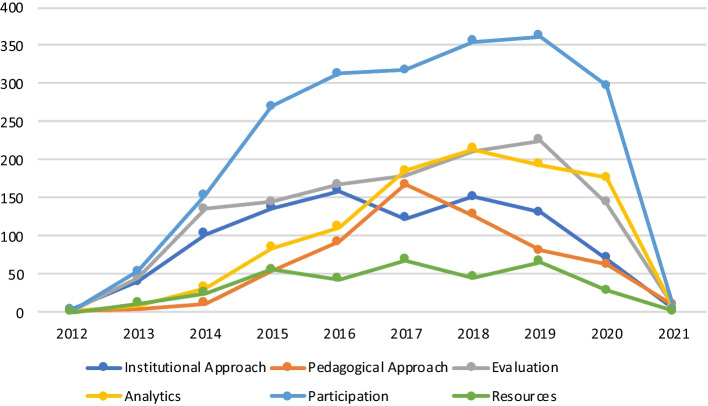
Thematic cluster temporal sequence

**Fig. 10:**
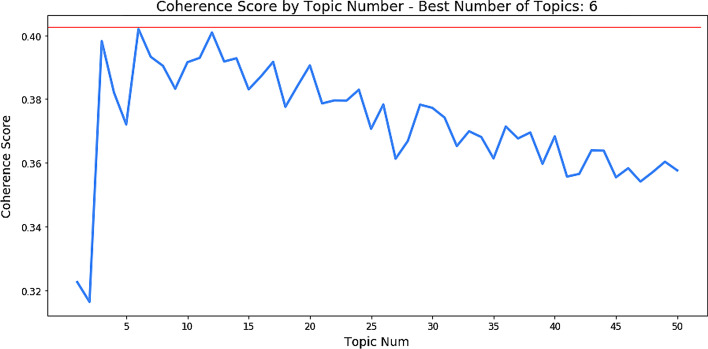
5–50 Clusters

Regarding the timeline of the production within each one of the thematic clusters, most have a similar trajectory to the general one. Figure 9 shows how the groups focused on institutional approaches, evaluation and participation concentrated the first interests in MOOCs literature until 2014. After 2015, the high growth rate is maintained in the participation group until 2019 and, with a much softer slope, in the evaluation group. It is important to remark that this analysis is only reliable until 2020, as the query was done in December 2020 (just a few papers from 2021 are included).

The delayed growth of the analytics and pedagogical approach groups is worth being considered. With the pedagogical approach, it had its peak in 2017, and with the analytics cluster, it continues growing until 2018 and then starts to decrease slowly. The institutional approach cluster and the one about resources have maintained a regular profile of publication since 2015.

From the authors' viewpoint, the other relevant question is how these papers and clusters have influenced the subsequent literature.

Understanding that a paper influences the subsequent literature when it is cited, the influence of each thematic cluster can be analyzed by looking at the percentage of papers of the cluster that have received at least one citation. As seen in Table 3, the most influencing cluster in literature has been the one regarding analytics, in which more than a quarter of the produced papers (27.55%) have been cited at least once. In the second place by percentage, we find the resources cluster, where 23.78% of papers have been cited at least once. But the cluster with the smaller impact is the pedagogical approach, where only 9.27% of the papers have been cited, followed by the institutional approach.

**Table 3:** MOOCs literature thematic Groups: indicators of subsequent influence

		Institutional approach		Pedagogical approach		Evaluation	Analytics		Participation			Resources
At least 1 citation in databases	N	140	57		268			281		476	83	
	%	15.09	9.27		21.15			27.55		22.23	23.78	
Over 100 citations	n	2	0		1			2		2	0	
	%	1.43	0		0.37			0.71		0.42	0	
Over 50 citations	N	3	0		5			7		5	2	
	%	2,14	0		1.87			2.49		1.05	2.41	
Over 10 citations	N	37	3		52			56		81	12	
	%	26.4	5.17		19.4			23.5		17	14.46	
*h*-index		20	5		21			22		25	11	

Looking at the number of citations by paper, the percentage of cited papers that have received over 50 citations along these years is very low (in the cluster of pedagogical approach is null), and the percentage of cited papers that received over 10 citations is still very small.

We can see that the cluster that received more citations by paper in percentage is the one focused on institutional approach, where 26.4% of the cited papers were cited over 10 times, followed by the cluster about analytics (23.5%). The cluster with fewer citations per paper is the one regarding pedagogical approach (5.17%) followed by resources, with just 14.46% of cited papers that received over 10 citations.

If we consider the *h*-index of each cluster, it gets even more interesting. Following the original definition of the *h-*index done by Hirsh (2005), we say that a group has index *h* if *h* of its *Np* papers has at least *h* citations each, and the other *(Np-h*) papers have no more than h citations each. The *h-*index has been considered as a good index to analyze scientific production because it measures the quantity and the impact of a set of literature -in the case of this study, of a thematic cluster- with only one figure (Alonso et al., 2020).

Here, for the less influent clusters (pedagogical approach and resources), the *h-*index reinforces the results obtained from the percentage of the number of citations. However, for the others, it remarks the importance of the thematic cluster regarding participation (the one with the biggest production), as it has the highest *h-*index without having the highest percentage of articles cited over 10 times, what also happens with the analytics cluster (with the second highest h-index).

## Conclusions and discussion

The main goal of this research was analyzing MOOC-related publications since the emergence of the term in the context of education with ML and VNA, offering a complementary and more extensive vision to previous literature reviews on MOOCs, looking at the whole picture of what has been published.

The study has shown the potential of ML and VNA techniques to automatize systematic literature review processes that otherwise would be much more cumbersome processes in terms of time and resources. These techniques are quantitative processes and require human knowledge in the field being analyzed to adequately interpret the results obtained by the automatic procedures. A good example of this was the discussion among experts about which clusters represented better MOOC research and how they could be named. The machine-learning's vision provides these studies with volumes of analysis that people cannot reach without efforts that are often beyond our reach, but the subjective analysis of delicate and complex issues, as in the case of education, requires levels of expertise and complexity that, at least for the moment, are still lacking in purely automatic approaches.

Concerning RQ1, we identified two divisions in clusters (6 clusters and 12 clusters) based on the coherence score of a range of models. After the expert discussion on the two classifications based on an educational perspective, the classification in 6 clusters was selected and further analyzed. The 6 clusters in the selected division refer to these topics: institutional approach, pedagogical approach, evaluation, analytics, participation and educational resources.

In the previous literature reviews (see Table 1), only 6 included thematic divisions or a tentative thematic classification of the most important topics on the field (Raffaghelli et al., 2015; Sa'don et al., 2014; Sangrà et al., 2015; Veletsianos & Shepherdson, 2016; Yousef et al., 2014; Zhu et al., 2018).

Out of the 6 clusters in this paper, just two were also identified in the manual reviews. The cluster called "institutional approach", that receives other titles (Business model, Policy and Instructional design, Institutional objectives, Consequences for the higher education system, MOOCs for institutional development, Context and impact, Higher education), but includes the same papers; and the "pedagogical approach" cluster that is identified in them all, also under other denominations (Design, Design for learning in MOOCs, Design focused, Instructional/MOOC design, Learning theories, Pedagogy, Testing pedagogical strategies, MOOC pedagogy). Sometimes, the latter is divided into two clusters (Raffaghelli et al., 2015; Yousef et al., 2014; Zhu et al., 2018). A thematic cluster about Evaluation is included in three reviews (Sa'don et al., 2014; Yousef et al., 2014; Zhu et al., 2018). Only two other reviews included something related to educational resources (Rafagghelli et al., 2015; Sa'don et al., 2014), but in both cases, they are mentioned as "technology" or "technological tools". Only the review carried out by Sangrà et al. (2015) identified a thematic cluster about the use of Learning analytics. Finally, even if the role of social media is understood in some previous reviews as a thematic cluster, the participation (the one in the present review) is not.

After the analysis, we consider that the opportunity of having a first automated approach to the clustering division complemented with an expert educational approach—provided by humans, helps the review not only to be relevant but also to be coherent from the educational viewpoint.

The RQ2 concerns the internal characterization of each of the 6 clusters. For that purpose, we used VNA and identified thematic subclusters within each topic based on the relationships between terms based on human expertise. This analysis allowed us to confirm the initial topic names/interpretation and supported a deeper understanding of each cluster's studies. Therefore, the combined work between machine and human in this task also gave good results.

In terms of relationships of relevance, impact, and influence between the thematic clusters (RQ3), we can see that the lowest production of MOOC papers is within the topics of pedagogical approach and educational resources, while participation and evaluation papers are the most frequent in the sample. However, the most cited papers are within analytics and resources, being pedagogical approach and institutional approach the ones with fewer citations.

These metrics results, as well as the size and contents of the pedagogical approach cluster, reinforce the absence of topics about education and pedagogy in the papers about educational technology, also pointed out by other authors (Bartolomé, et al., 2018; Zawacki-Richter, et al., 2019). This situation calls for educators to be involved in the MOOC discussion from a more pedagogical point of view, which also should improve educational practice in an evidence-based way. Also, the need to connect educators working in the field, getting to know what others do—and citing previous work—, would also build upon pedagogical work and give more emphasis on the pedagogical aspect of MOOCs, instead of other topics that are not the core of educational technology.

The percentage of cited papers with over 50 citations—and even 10 citations—is very low for all clusters, and, considering the *h*-index, the order is different for the most cited clusters, as participation (the most popular) and analytics are the ones with the highest *h*-index value and are not the ones with the highest percentage of cited articles. Interestingly, the curve of the evolution of research in the different groups seems to reflect the Gartner hype for technology (Gartner, 2018) and suggests that after the innovation trigger in 2012, MOOC research reached the peak of inflated expectations in 2018–2019. Now it has started a phase that may lead to the trough of disillusionment, before (presumably) reaching the slope of enlightenment. It remains to be seen how the Covid-19 pandemic has affected MOOC research and this curve as in other educational areas (Bond, et al., 2021).

Finally, the RQ4 has identified the missing MOOC research. From our findings, there is a clear need for more influential MOOC research from a pedagogical perspective. For instance, there is almost no research regarding pedagogical models and instructional and learning theories applied to MOOCs. Also, there is a strong need to move forward in the research conducted on the topics of participation and pedagogical approach and go beyond aspects that quantify participation. But some aspects are overlooked by the MOOC research, which is focused on the technological aspects. For instance, so far, from the visualizations we have obtained, and taking into account the threshold of words configured in the system, few or null research can be observed that addresses social issues such as digital divide, data privacy, ethics, intercultural aspects or internationalization, which coincides with missing or scarce work and discussion in educational technology around these topics (Kimmons & Rosenberg, 2022).

As a final remark and considering that 74 reviews have been published in the last 10 years about MOOCs, it is time to accumulate and aggregate data to consolidate knowledge about e-learning using meta-analysis techniques. Meta reviews will support the extraction of more robust results and conclusions that inform policy and develop evidence-based practice.

Finally, it should be noted that, while the perspective of this paper was to analyze what we have wanted to research and explore over the years about MOOCs, the results and conclusions that these data show us—especially those that talk about the citation of articles related to social issues—speak clearly about "What we do not seem to want to know about MOOCs" and may require some more profound future analysis of our motivations in the field.

In addition, studies such as the one presented in this paper highlight the importance of approaching research questions from increasingly hybrid perspectives that take advantage of the potential offered by technologies and, at the same time, highlight the immense contribution that human expertise can make in this regard. However, such approaches have been little explored and only vaguely documented, which underlines the importance of developing the methodologies of these and other analyses in more detail and depth and even of exploring new forms of methodologies more rigorously. This effort must be made in studies such as this one, where the object of study is the published literature, and in other methodological articles where the object of study is the methodology itself.

## Data Availability

The data presented in this study are available on request from the corresponding author. The data are not publicly available due to privacy regulations.

## Appendix A. Coherence score of the models

See Fig. 10.

## Appendix B. Classifications of six and twelve groups

Six groups:

- Cluster 0: education university course development technology platform institution quality country world opportunity paper challenge access MOOC
- Cluster 1: teaching mode college classroom reform model method teacher application ability resource computer learning curriculum effect
- Cluster 2: student course assessment performance study datum peer result feedback engagement group programming forum rate activity
- Cluster 3: learner course datum learning engagement study rate behaviour forum self-interaction completion dropout analysis motivation
- Cluster 4: learning design teacher research study environment participant process development tool approach experience framework project community
- Cluster 5: video lecture content style user feature resource course production quiz material time platform topic behaviour.

Twelve groups:

- Cluster 0: education university technology development institution quality opportunity innovation challenge country world distance access MOOC internet
- Cluster 1: teaching mode college classroom reform method application computer curriculum ability resource advantage model effect practice
- Cluster 2: student course study group performance engagement result motivation class face completion activity university rate satisfaction
- Cluster 3: learner engagement study motivation course completion rate self-behaviour factor interaction activity datum time group
- Cluster 4: teacher training development school knowledge project service skill competence face ICT language program experience classroom
- Cluster 5: video lecture content style production quiz feature material time topic behaviour text resource concept classroom
- Cluster 6: model datum prediction feature dropout behaviour performance method user algorithm analysis analytic research problem rate
- Cluster 7: learning environment self-process technology tool strategy knowledge community activity resource face approach experience language
- Cluster 8: course platform user content participant paper knowledge experience number resource language web information people tool
- Cluster 9: assessment peer feedback evaluation grading quality assignment programming grade review question task approach submission tool
- Cluster 10: forum discussion interaction post thread network participant community instructor analysis participation question topic activity content
- Cluster 11: design research study framework participant gamification principle project analysis evaluation literature result approach development review

## Abbreviations

MOOC: Massive open online courses
ML: Machine learning
RQ: Research question
SNA: Social network analysis
VNA: Visual network analysis
